# Children’s Ailments and Household Preventitives

**Published:** 1896-01

**Authors:** 


### Children's Ailments and Household Preventives.

Some ailments of children are like a simple mechanical puzzle—obscure, complicated, alarming in their symptoms, very troublesome if mismanaged, but in their cause and explanation simplicity itself. Last week a boy was taken ill from the effects, as was supposed, of a heavy dinner. Thanks to the guiding symptom of pain the true seat of his disorder was quickly discovered, and the rectum was found to be tightly packed with the broken but unmasticated fragments of nuts, a meal of which he had recently consumed on his own account. An anæsthetic and forceps relieved him of his load, the more usual means having failed to do so. A mouth full of decayed teeth showed how, in his case, mastication was well nigh impossible. The danger allowing so much rough and hard matter in a state quite incapable of digestion to traverse the length of the intestinal tract or to become impacted at some point in it is such as even an unprofessional mind can appreciate. This case, unique perhaps in actual particulars, is one of many, some of them as clear, and some obscure in character, but all capable of much mischief and misunderstanding, which bear one common accusation—indiscretion in diet. Now it is a poisonous ice cream, now a stale fruit, now a mass of broken nuts. The inevitable question arises—Who is responsible? And its answer will usually acquit the salesman if he be a careful and fair dealer. Child and parent, therefore, must divide the blame, if any, and of the two the more intelligent will naturally have the larger share. Such cases as that above quoted should at all events impress upon parents the lesson, too little regarded, that failure to warn, and to prevent if needful, by active measures, the heedless indiscretions of their children may at any time induce, perhaps by very simple means, an illness of the greatest gravity.—*London Lancet, August* 24.

---

A MIXTURE of chloroform (ten parts), ether (fifteen parts), and menthol (one part), used as a spray, is recommended as an excellent and prompt means of obtaining local anæsthesia, lasting for about five minutes.—*Boston Med. and Surg. Journal.*

